# Reduced reproductive performance associated with warmer ambient temperatures during incubation in a winter‐breeding, food‐storing passerine

**DOI:** 10.1002/ece3.2864

**Published:** 2017-03-23

**Authors:** Shannon Whelan, Dan Strickland, Julie Morand‐Ferron, D. Ryan Norris

**Affiliations:** ^1^ Department of BiologyUniversity of OttawaOttawaONCanada; ^2^ 1063 Oxtongue Lake RoadDwightONCanada; ^3^ Department of Integrative BiologyUniversity of GuelphGuelphONCanada

**Keywords:** brood size, brooding, corvid, food storage, phenology, timing of breeding

## Abstract

Timing of reproduction can influence individual fitness whereby early breeders tend to have higher reproductive success than late breeders. However, the fitness consequences of timing of breeding may also be influenced by environmental conditions after the commencement of breeding. We tested whether ambient temperatures during the incubation and early nestling periods modulated the effect of laying date on brood size and dominant juvenile survival in gray jays (*Perisoreus canadensis*), a sedentary boreal species whose late winter nesting depends, in part, on caches of perishable food. Previous evidence has suggested that warmer temperatures degrade the quality of these food hoards, and we asked whether warmer ambient temperatures during the incubation and early nestling periods would be associated with smaller brood sizes and lower summer survival of dominant juveniles. We used 38 years of data from a range‐edge population of gray jays in Algonquin Provincial Park, Ontario, where the population has declined over 50% since the study began. Consistent with the “hoard‐rot” hypothesis, we found that cold temperatures during incubation were associated with larger brood sizes in later breeding attempts, but temperatures had little effect on brood size for females breeding early in the season. This is the first evidence that laying date and temperature during incubation interactively influence brood size in any bird species. We did not find evidence that ambient temperatures during the incubation period or early part of the nestling period influenced summer survival of dominant juveniles. Our findings provide evidence that warming temperatures are associated with some aspects of reduced reproductive performance in a species that is reliant on cold temperatures to store perishable food caches, some of which are later consumed during the reproductive period.

## 1 Introduction

The timing of an individual's reproductive efforts can have important fitness consequences. Early breeding individuals tend to have higher reproductive success than late breeders (Daan, Dijkstra, Drent, & Meijer, 1988; Green & Rothstein, 1993; McKellar, Marra, & Ratcliffe, 2013; Réale, Berteaux, McAdam, & Boutin, 2003; Reed et al., 2006). Early breeders are more likely to initiate breeding again after a failed attempt (Pakanen, Rönkä, Thomson, & Koivula, 2014) and have a higher probability of breeding multiple times in a season (Böhning‐Gaese, Halbe, Lemoine, & Oberrath, 2000; Gil‐Delgado, Marco, Paredes, & Vives‐Ferrándiz, 2005). Early breeders also tend to produce more offspring (Daan et al., 1988; Rowe, Ludwig, & Schluter, 1994) that are in better condition (e.g., Green & Rothstein, 1993; Stier et al., 2014). Furthermore, offspring from early reproductive attempts are more likely to survive (Naef‐Saenzer, Widmer, & Nuber, 2001) and recruit into the breeding population (Descamps, Boutin, Berteaux, & Gaillard, 2006; Green & Rothstein, 1993), increasing the fitness benefits for early breeders.

Although early reproduction can confer fitness benefits, climatic conditions during offspring development can also influence reproductive success regardless of when individuals begin reproduction. Incubation of eggs and the care (brooding and feeding) of nestlings are energetically expensive behaviors, (Sanz & Tinbergen, 1999; Williams, 1996), and this energetic expenditure can be influenced by food availability and ambient temperature (e.g., Tinbergen & Dietz, 1994). In extreme cases, parents will abandon a nest due to poor weather conditions (Elkins, 2010) or insufficient food (Anderson, 1989). Furthermore, ambient temperature is often linked to food availability, which in turn influences the ability of parents to successfully rear offspring (e.g., van Noordwijk, McCleery, & Perrins, 1995; Reed, Jenouvrier, & Visser, 2013). Parents may increase foraging efforts to compensate for low food availability or poor weather during the incubation (Bentzen, Powell, Phillips, & Suydam, 2010) and nestling periods (Johnson & Best, 1982). Consequently, both parents and offspring may be at a higher risk of predation (DuRant, Hopkins, Hepp, & Walters, 2013), or reduce incubation constancy, which can lower offspring condition and survival (Bentzen et al., 2010; DuRant et al., 2013). Although there is evidence that timing of breeding and climatic conditions during incubation and nestling periods can both affect reproductive outcomes, little is known about how these two factors may interact to influence reproductive success in birds.

**Figure 1:**
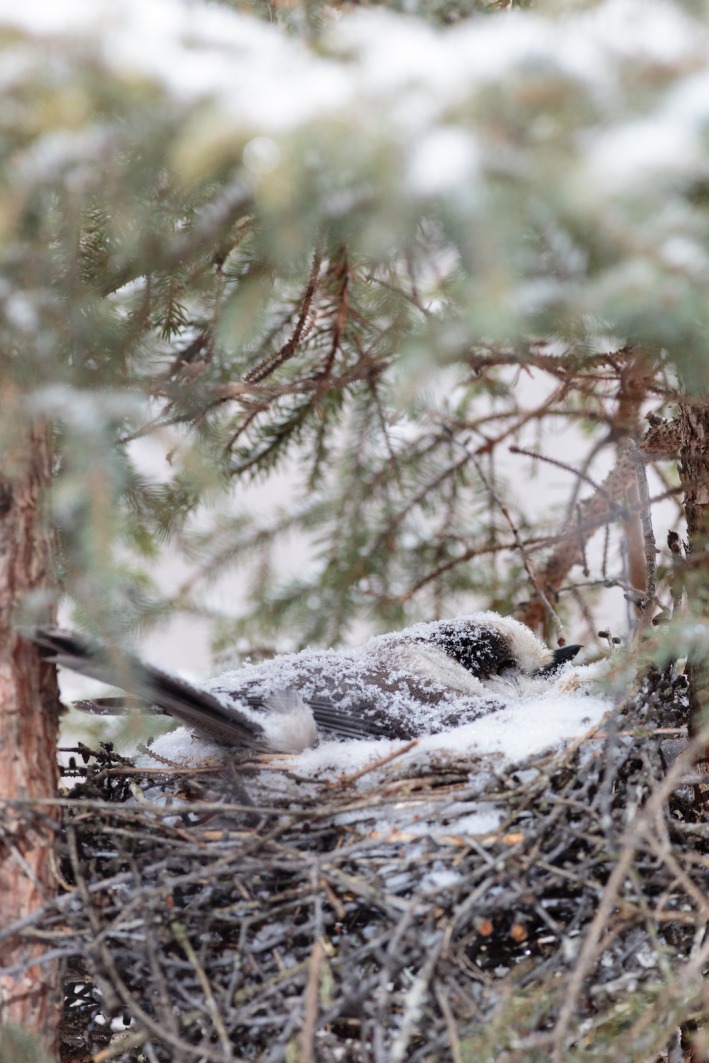
Female gray jay incubating during winter in Algonquin Provincial Park, ON. Photo credit: Brett Forsyth

Our objective was to determine whether ambient temperatures during incubation and nestling periods modulate the effect of timing of breeding on reproductive performance in a population of gray jays (*Perisoreus canadensis*, Figure 1) breeding in Algonquin Provincial Park, Ontario. Gray jays are year‐round residents of North American boreal and subalpine forests that store perishable food (e.g., berries, mushrooms, invertebrates, vertebrate carrion) during late summer and autumn (Strickland & Ouellet, 2011). Pairs initiate breeding in mid‐ to late winter, when temperatures are typically below freezing and fresh food resources are scarce (Strickland & Ouellet, 2011). In Algonquin Park, the dominant juvenile of a brood ejects its subordinate siblings from the natal territory in June, ca six weeks after fledging, and typically stays with its parents for the following year, usually dispersing in its second summer (Strickland, 1991). Subordinate juveniles forced to disperse from the natal territory by the dominant juvenile likely have poor first‐summer survival but can recruit with unrelated gray jay pairs that were unsuccessful in rearing their own offspring (Strickland, 1991), or attempt to breed.

Previous work on the Algonquin population linked warming autumn temperatures to a long‐term population decline and proposed that warm temperatures in late autumn degrade the quality of perishable food stores on which gray jays rely during winter reproduction (the “hoard‐rot” hypothesis; Waite & Strickland, 2006). Using an experimental approach, Sechley, Strickland, and Norris (2015) found that cold temperatures better preserved artificial food caches than warm temperatures, providing further indirect evidence that warm temperatures may degrade gray jay food stores. The timing of breeding can also influence gray jay reproductive success (Whelan, Strickland, Morand‐Ferron, & Norris, 2016). Early breeders are more likely to successfully raise young to 11‐day old and have a dominant juvenile that survives until fall (Whelan et al., 2016). Timing of reproduction and brood size is food limited in this population; females advance laying and have larger brood sizes when food supplemented (Derbyshire, Strickland, & Norris, 2015). Although there is strong evidence that laying date influences reproductive success in Algonquin gray jays (Whelan et al., 2016), and warmer autumn temperatures are associated with reduced clutch size (Waite & Strickland, 2006), we do not know whether temperatures during the incubation and nestling periods alter the fitness costs of late reproduction.

We examined an extension of the “hoard‐rot” hypothesis (Waite & Strickland, 2006) to test whether, through their influence on cached food, ambient temperatures during the incubation and nestling periods might affect: (1) brood size (i.e., number of nestlings at banding) or (2) the summer survival of dominant juveniles. Specifically, we hypothesized that the costs of later laying would be increased (lower reproductive performance) during warm incubation and nestling periods under the assumption that warmer temperatures do not preserve cached food as well as colder temperatures. In contrast, there may be little or no cost to laying later when temperatures are cold during the incubation and nestling periods. Following this hypothesis, we predicted an interaction between laying date and incubation/nestling period temperatures such that, at later laying dates, brood sizes would be larger under colder temperatures but that temperature would have no effect on brood size at earlier laying dates. Similarly, we predicted that summer survival would be higher for dominant juveniles that resulted from nesting attempts occurring at colder temperatures than it would be for those resulting from nesting attempts occurring at warmer temperatures.

The effect of ambient temperature on reproductive performance could also depend on which phase of offspring development is examined. The female is largely dependent on male provisioning during incubation and the first week of the nestling period but subsequently takes on an increasing role in foraging for the nestlings, albeit almost always in company with the male (Strickland & Ouellet, 2011). Furthermore, important fresh food resources often become available when snowmelt exposes the forest floor during the nestling period (D.S., pers. obs.). As the incubation and nestling periods differ in their demands for, and availability of, food resources, we tested for the effect of ambient temperatures separately for each of these time periods (although for practical reasons [see below], we were able to consider only the early part of the nestling period).

## 2 Methods

### 2.1 Study system

We studied a marked population of gray jays in southern Algonquin Provincial Park, Ontario (45°N, 78°W) from 1978 to 2015. Individuals were marked with a unique combination of color bands and a numbered aluminum band issued by Canadian Wildlife Service. In annual fall censuses, we estimated age of immigrants into the study area as either juvenile (first year) or adult (second year or older) by examining rectrix shape (Strickland & Ouellet, 2011). Breeding pairs in this population typically initiate breeding between late February and March, when ambient temperatures were below 0°C. Gray jay pairs rely on stored food for winter survival and, at least in part, during reproduction (Sechley, Strickland, & Norris, 2014; Strickland & Ouellet, 2011). Only females incubate, but males provision females during the incubation and early nestling periods (Strickland & Ouellet, 2011).

### 2.2 Monitoring of reproduction

We located nests through behavioral observations and revisited them every 2–5 days to determine laying date and reproductive success. We considered laying date to be the midpoint between the earliest and latest possible dates of clutch initiation, and calculated *relative laying date* as the female's laying date relative to other females breeding in the population that year (Lewis, Nussey, Wood, Croxall, & Phillips, 2012; Reed et al., 2009; Whelan et al., 2016). Female gray jays sit from their first egg but usually initiate incubation only when the clutch is complete, resulting in an apparent incubation period of 20 days for a typical three‐egg clutch (i.e., 18 days of true incubation plus 2 days from the laying of the first egg to the laying of the third egg; Strickland & Ouellet, 2011). We banded nestlings approximately 11 days after the estimated hatching date (laying date of first egg + 20 days of incubation). Given the height of most nests and the risk of causing premature departure by nestlings if nest contents were checked in the late nestling period, we were unable to positively determine brood size at fledging and therefore used the number of nestlings at banding as our measure of brood size. The following fall, we conducted a population census and determined whether a dominant juvenile survived the summer.

### 2.3 Ambient temperature during incubation and nestling periods

We obtained historical temperature records from Environment Canada for two weather stations: one operated west of the study area (Dwight, Ontario, 45°23′N 78°54′W) from the beginning of the study period until 2005 and the second (Algonquin Park East Gate, Ontario, 45°32′N 78°16′W) began operation within the study area in 2004. We used reduced major axis regression for the period of overlapping station operation to estimate winter temperatures within the study region from 1978 to 2004 by transforming mean daily temperatures from the western weather station with the regression equation (see Whelan et al., 2016). We calculated *mean incubation temperature* as the mean of mean daily temperatures between egg‐laying and the estimated hatching date, and *mean nestling period temperature* as the mean of mean daily temperatures between the estimated hatching date and 11‐day posthatch.

### 2.4 Food supplementation

Food supplementation by park visitors has been shown to advance laying date and increase clutch and brood sizes in this study population (Derbyshire et al., 2015), thus we accounted for level of food supplementation on territories in all models. Territories were classified as having a low (little or no public access to territory), medium (public feeding during autumn only), or high (public feeding during autumn and winter or permanent feeder on territory) level of supplementation (see also Derbyshire et al., 2015; Whelan et al., 2016).

### 2.5 Final dataset

For our analyses, we used all first nest records of marked pairs for which we observed laying dates and brood size. We included failed breeding attempts for which we obtained laying dates (i.e., brood size = 0) and excluded breeding attempts of pairs that were experimentally food supplemented in 2013 and 2014 (*N* = 20; Derbyshire et al., 2015). The final dataset for models of brood size included 597 nest records from 175 females monitored between 1978 and 2015. The final dataset for models of dominant juvenile summer survival was a subset of the brood size dataset (*N* = 589 nest records, 174 females, 38 years).

### 2.6 Statistical analyses

We examined variation in brood size and dominant juvenile summer survival using fixed effects of relative laying date, female age, food supplementation, mean ambient temperature (during the incubation period and during the first 11 day of the nestling period), and an interaction between relative laying date and mean ambient temperature. We fit generalized linear mixed models of brood size (Poisson distribution) and dominant juvenile summer survival (binomial distribution) with maximum likelihood (Laplace approximation) using the statistical package *lme4* (Bates, Mächler, Bolker, & Walker, 2015) in *R* (version 3.2.3, R Core Team 2015). Random effects of female identity and year were included in all models. All continuous predictor variables were standardized by grand mean centering and dividing by one standard deviation. We used variance inflation factors (VIF) to test for collinearity between all predictor variables for all models (Zuur, Ieno, Walker, Saveliev, & Smith, 2009). For models of brood size, we tested for overdispersion.

We used Akaike's Information Criterion adjusted for small sample size (AIC_C_) for model selection (*R* package *MuMIn*; Bartoń, 2016). Our null model included variables previously linked to brood size in this population (i.e., female age, level of food supplementation, and relative laying date). For each temperature window (incubation, early nestling period), we included one model with a main effect of the temperature variable and a second model including an interaction term between the temperature variable and relative laying date. We also constructed models of dominant juvenile summer survival with the same model structure stated above for brood size, and compared the five models with AIC_C_ model selection.

## 3 Results

Mean incubation temperatures ranged from −11.8 to 10.8°C (mean = −0.7°C ± 3.6 *SD*), and early nestling period temperatures ranged from −8.3 to 13.8°C (mean = 3.2°C ± 3.6 *SD*). Mean ambient temperatures did not increase significantly over the study period (incubation: slope = 0.0051°C/a, *F*_1,36_ = 0.024, *p* = .88, *R* ^2^ = .0007; brooding: slope = 0.0076°C/a, *F*_1,36_ = 0.060, *p* = .81, *R* ^2^ = .002). Brood size varied from zero to five nestlings (mode = 3) and was not overdispersed (χ^2^ = 569, *df* = 588, *p* = .71). Female age ranged from 1 to 16 year (mean = 4.7 year ± 3.2 *SD*). VIFs of fixed predictors were <3 for all models and, therefore, within an acceptable range of collinearity (Zuur et al., 2009).

**Figure 2:**
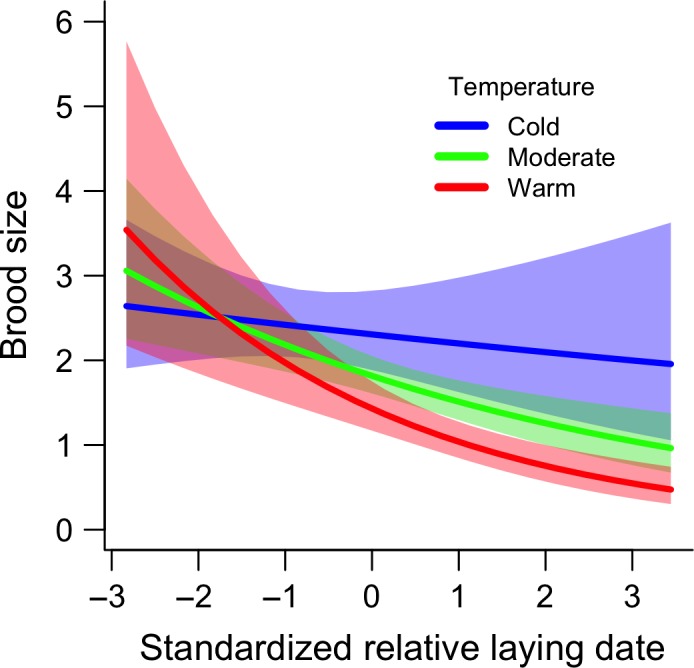
Relative laying date and mean ambient temperature during incubation had an interactive effect on brood size. Model predictions are derived from the top model from AIC_C_ model selection presented in Table 1. We generated model estimates at three discrete temperatures to visualize the interaction between two continuous fixed effects (cold = −6.2°C, moderate = −1.0°C, and warm = 5.2°C). Shaded areas represent 95% confidence intervals

The top model for brood size (*W* = 0.93) included an interactive effect between relative laying date and ambient temperature during incubation (Table 1). The AIC_C_ value of next best model was 6.41 greater than the AIC_C_ value of the top model (Table 1). For the top model, brood size was larger for breeding attempts initiated at earlier relative laying dates than later dates but, consistent with the hoard‐rot hypothesis, we observed larger brood sizes among late nests incubated at colder temperatures than late nests that were incubated at warmer temperatures (Figure 2; Table 3).

**Table 1:** Results of AIC_C_ model comparisons, modeling brood size in response to multiple fixed predictors and random effects of year and female identity. All models included in the model selection are shown (*N* = 175 females, 597 nest records, 38 years)

Model	Fixed effect terms	AIC_C_	∆AIC_C_ a	*W* b	ER^c^
1	Relative laying date + incubation temperature + female age + food supplementation + relative laying date × incubation temperature	1996.8	0	0.927	
2	Relative laying date + incubation temperature + female age + food supplementation	2003.2	6.41	0.038	24.4
3	Relative laying date + nestling period temperature + female age + food supplementation + relative laying date × nestling period temperature	2004.8	8.00	0.017	54.5
4	Relative laying date + nestling period temperature + female age + food supplementation	2005.8	9.01	0.010	92.7
5	Relative laying date + female age + food supplementation	2006.3	9.56	0.008	115.9

a Difference between AIC_C_ value of top‐ranked model and given model.

b Akaike weight.

c Evidence ratio.

In contrast to brood size, there was not a clear top model for summer survival of dominant juveniles (Table 2). All models tested were within 5 AIC_C_ of the best‐fitting model. However, the null model, which did not include ambient temperature during the incubation or early nestling periods, was the best‐fitting model. We conducted full model averaging on the two top models (∆AIC_C_ < 2). In contrast to the brood size results, there was little support for an effect of ambient temperature during the incubation or early nestling periods on dominant juvenile summer survival. Although a main effect of ambient temperature during the early nestling period was included in one of the top models (model 2, Table 2), the 95% confidence interval of ambient temperature during the early nestling period included zero after model averaging (Table 3).

**Table 2:** Results of AIC_C_ model comparisons, modeling summer survival of a dominant juvenile in response to multiple fixed predictors and random effects of year and female identity. All models included in the model selection are shown (*N* = 174 females, 589 nest records, 38 years)

Model	Fixed effect terms	AIC_C_	∆AIC_C_ a	*W* b	ER^c^
1	Relative laying date + female age + food supplementation	723.9	0	0.435	
2	Relative laying date + nestling period temperature + female age + food supplementation	725.0	1.11	0.250	1.74
3	Relative laying date + incubation temperature + female age + food supplementation	726.0	2.05	0.156	2.79
4	Relative laying date + nestling period temperature + female age + food supplementation + relative laying date × nestling period temperature	726.8	2.89	0.102	4.26
5	Relative laying date + incubation temperature + female age + food supplementation + relative laying date × incubation temperature	728.0	4.08	0.056	7.77

a Difference between AIC_C_ value of top‐ranked model and given model.

b Akaike weight.

c Evidence ratio.

**Table 3:** Parameter estimates for top model for brood size (model 1, Table 1) and full model averaged parameter estimates for summer survival of a dominant juvenile (AIC_C_ < 2: models 1 and 2, Table 2). Levels of “food supplementation” were as follows: low (reference category), M, medium; H, high (see methods for details). We report 95% confidence intervals

Term	Brood size			Dominant juvenile survival (Y/N)		
	Estimate ± SE	2.5% CI	97.5% CI	Estimate ± SE	2.5% CI	97.5% CI
Intercept	0.67 ± 0.061	0.55	0.79	−0.48 ± 0.17	−0.81	−0.15
Relative laying date	−0.18 ± 0.049	−0.28	−0.084	−0.45 ± 0.13	−0.70	−0.20
Ambient incubation temperature	−0.15 ± 0.051	−0.25	−0.050	–	–	–
Ambient nestling period temperature	–	–	–	−0.046 ± 0.10	−0.24	0.15
Female age	0.056 ± 0.031	−0.0048	0.12	−0.0084 ± 0.095	−0.19	0.18
Food supplementation						
M	−0.063 ± 0.076	−0.21	0.086	−0.28 ± 0.22	−0.71	0.15
H	−0.15 ± 0.083	−0.31	0.013	−0.79 ± 0.26	−1.30	−0.28
Relative laying date × ambient incubation temperature	−0.087 ± 0.031	−0.15	−0.026	–	–	–

## 4 Discussion

Our results provide, to the best of our knowledge, the first evidence that laying date and ambient temperatures during incubation can interactively influence brood size in birds. This finding is consistent with Waite and Strickland's (2006) “hoard‐rot” hypothesis, which reasons that warm autumn temperatures could degrade food caches, resulting in a food limitation in the subsequent breeding season. Poor‐quality food may require females to increase foraging trips to meet their nutritive needs, thus decreasing incubation constancy (Bentzen et al., 2010) and increasing the risk of nest and adult predation (DuRant et al., 2013). Furthermore, females with degraded food stores could deplete endogenous reserves more rapidly than females with access to high‐quality food, and depletion of endogenous reserves is associated with nest desertion (Yorio & Boersma, 1994). In gray jays, late nesting or inexperienced incubating females sometimes vocally beg from their nests and/or leave their nests to pursue their mates. This suggests inadequate food stores or an inadequate mate, leading in either case to inadequate endogenous reserves, a prelude to the death of individual embryos and/or nest abandonment. An alternative hypothesis for an association between warm temperatures during incubation and reduced reproductive success is that nestlings and parents overheat (e.g., *Falco naumanni*, Serrano, Tella, & Ursuia, 2005; *Falco tinnunculus*, Charter, Izhaki, Bouskila, & Leshem, 2007). However, gray jays incubate and brood in winter conditions, making hyperthermia an unlikely mechanism for observing smaller brood sizes at warmer temperatures.

In contrast, we did not find evidence that cold temperatures during brooding were associated with higher summer survival of dominant juveniles. One reason for this might be because this metric of reproductive success does not capture total juvenile recruitment. Fluctuations in temperature may have a greater effect on the survival of ejected juveniles because they are likely subdominant and in poorer condition. Unfortunately, we were not unable to track or estimate the survival of ejected juveniles as most of them disperse out of our study area. It is possible that the recruitment of ejectees on the territories of unrelated breeders may be a significant contributor to reproductive success (Strickland 1991; Whelan et al., 2016).

Further work is needed to determine whether degradation of food stores underlies the interactive effect between ambient temperature and timing of reproduction on gray jay brood size. It is important to note that, after nest failure, renesting attempts are quite successful (23 of 38 renests observed during the study period produced nestlings at 11 day). However, as only early breeders (i.e., those with previous experience [Whelan et al., 2016] or with access to supplemental food [Derbyshire et al., 2015]) can renest after a failed attempt, it may be that these individuals still have sufficient food stores despite their rapidly diminishing quality. Additionally, the hypothesized negative impact of increased hoard‐rot resulting from warmer late season temperatures could be offset by increased availability of invertebrates later in the breeding season. To improve our understanding of the role of temperature in the preservation of gray jay food caches and reproductive success, observational studies could compare cache retrieval trips of females at different times during the breeding season and determine whether ambient temperatures during incubation predicts the duration of time females spend retrieving caches. If caches do indeed degrade with warm temperatures in late winter and spring, we expect females incubating at warmer temperatures late in the season will make more cache retrieval trips than females incubating earlier in the season or at colder temperatures. To experimentally determine whether food caches degrade more at warm temperatures late in the breeding season, artificial food caches could be deployed along a temperature gradient (see Sechley et al., 2015) during late winter and spring and retrieved at different time points.

Interestingly, warming ambient temperatures could produce opposing effects on reproductive performance of gray jays. In this study, we found that ambient temperatures after laying (during incubation) have the opposite effect on reproductive performance compared to temperatures prior to laying. Female gray jays lay earlier when they experience warmer temperatures before laying, possibly because temperature imposes a physiological limitation on timing of reproduction, and early reproduction relative to other breeders in the population is positively associated with reproductive success (Whelan et al., 2016). However, despite the wintry conditions in which gray jays breed, we did not find evidence of a cost to incubating at cold temperatures. Instead, we found that warm temperatures during incubation were associated with smaller brood sizes later in the breeding season but, early in the breeding season, brood sizes were similar across incubation temperatures. It is possible that only pairs in good condition with large amounts of stored food were capable of breeding early, and their large food stores buffered them from effects of temperature. Alternatively, the benefit of cold ambient temperatures with respect to food storage may exceed the thermoregulatory costs during incubation in gray jays. In contrast, several studies have found that reproductive success increased with ambient temperatures in the incubation and nestling periods (Beck, Hopkins, Jackson, & Hawley, 2015; Chausson, Henry, Almasi, & Roulin, 2014; Hallinger & Cristol, 2011). However, these studies did not test whether the effect of temperature on reproductive success depended on timing of reproduction. Further studies should test for an interaction between timing of breeding and incubation temperature in a species that does not rely on cached food during the breeding season. In such a system, thermoregulatory costs of cold ambient temperatures during brooding could be offset by increased food availability (e.g., invertebrates) later in the breeding season.

Population declines have been documented in two areas along the southern limit of gray jay range (Menebroeker, Anich, Ogle, & Anich, 2016; Waite & Strickland, 2006), potentially due to warming temperatures associated with climate change (Waite & Strickland, 2006). Our findings bolster the evidence that a warming climate, specifically late winter temperatures, may negatively impact reproductive success in gray jays. Warming temperatures could thus lead to range loss at the southern edge of gray jay distribution, a well‐documented pattern observed in several taxa (Chen, Hill, Ohlemüller, Roy, & Thomas, 2011). We did not find evidence that ambient temperatures during the incubation or nestling periods have increased over time in the Algonquin population. However, warming temperatures during these stages of nesting could potentially negatively affect reproductive success in other gray jay populations, or with future climate change.

We found evidence that climatic conditions during incubation can influence the relationship between timing of breeding and brood size. Although gray jays breed in winter, we found no evidence that colder temperatures negatively impacted reproductive output. Indeed, colder temperatures were associated with larger brood sizes in the late breeding season, potentially because cold temperatures better preserve food caches that gray jays utilize during the breeding season. Although previous studies of other birds have indicated a positive link between ambient temperatures during incubation and reproductive success, we found, in the gray jay, further evidence consistent with an opposite and novel, indirect effect of temperature on reproductive performance.

## Conflict of interest

None declared.
